# Influence of diet quality on nutritional status of school-aged children and adolescents in Zanzibar, Tanzania

**DOI:** 10.1371/journal.pone.0293316

**Published:** 2023-10-20

**Authors:** Fatma Ali Said, Ahmed Gharib Khamis, Asha Hassan Salmin, Shemsa Nassor Msellem, Kombo Mdachi, Ramadhani Noor, Germana Leyna, Geofrey Joseph Mchau

**Affiliations:** 1 Department of Preventive Services and Health Promotion, Ministry of Health Zanzibar, Zanzibar, Tanzania; 2 Zanzibar Agricultural Research Institute, Zanzibar, Tanzania; 3 United Nations Childrens Fund, Zanzibar, Tanzania; 4 Office of the Chief Government Statistician, Zanzibar, Tanzania; 5 United Nations Childrens Fund, Addis Ababa, Ethiopia; 6 Tanzania Food and Nutrition Centre, Dar-es-salaam, Tanzania; Federal University of Agriculture Abeokuta, NIGERIA

## Abstract

**Background:**

Malnutrition among young children and adolescents poses a serious health challenge in developing countries which results in many health problems during adulthood. Poor diet quality is known as the root cause of malnutrition which is caused by unhealthy food choices and bad eating habits among young children and adolescents. However, limited evidence is available on diet quality and its association with nutrition status among young children and adolescents in Zanzibar. This study examined the diet quality and its relationship with the nutritional status of school-aged children and adolescents in Zanzibar.

**Methods:**

Data for this study was obtained from the cross-sectional survey of School Health and Nutrition (SHN) conducted in Zanzibar. The survey recruited children aged 5–19 years from 93 schools in Zanzibar. A seven-day food frequency questionnaire (FFQ) was used to assess dietary intake. Prime Dietary Quality Score (PDQS) consisted of 21 food groups was then constructed to assess the diet quality of school-aged children and adolescents. Body mass index (BMI-for-age Z-score) was used as the indicator of nutrition status. Both linear and logistic regression analysis techniques were used to determine the associations between BMI and PDQS.

**Results:**

A total data of 2,556 children were enrolled in the survey. The prevalence of thinness was 8.1%, normal 82.1%, overweight 7.2% and obesity 2.6%. The mean (SD) PDQS score was 18.8 (3.2) which ranged from 8 to 33. Consumptions of green leafy vegetables (49.3%), yellow or red fruits (37.8%), legumes (38.3%), fish (36.3%), and vegetable oil (31.5%) were up to three times per week, whereas consumptions of white vegetables (77.3%), cooked vegetables (32.6%), citrus fruits (66.8%), other types of fruits (66.2%), nuts (46.4%), poultry (49.6%), whole grains (61%) and eggs (67.8%) were less than once per week. In terms of unhealthy foods, eating fried foods was reported by 26.3% up to three times per week, and 31.5% reported consuming sweets and ice cream up to three times in the past week. High PDQS was significantly associated with a reduction in BMI of children (p< 0.005). For each unit increase in the consumption of unhealthy foods such as fried foods, cooked vegetables and refined grains there is a significant increase in BMI. The odds of being obese decrease significantly as diet quality increases from the first to third quintile of PDQS (AOR = 0.2, 0.04–0.89 95% CI, *p* = 0.035).

**Conclusion:**

Consumption of high quality diet was found to be associated with a reduction in excessive weight among school-aged children and adolescents in Zanzibar. There is a need for interventions targeting to reduce unhealthy food consumption in school environment. Further research should be conducted to assess diet quality using PDQS among young children and adolescents.

## Background

Malnutrition including all forms of undernutrition, overnutrition and micronutrients deficiencies poses a serious challenge in low and middle-income countries (LMICs) [1]. Various manifestations of child malnutrition have been consistently demonstrated, and in turn result in serious health problems that affect growth and development of children and adolescents [2]. Undernutrition may lead to underweight, poor performance at school, poor general health and less economic productivity [3]. While overnutrition may contribute to non-communicable diseases such as hypertension, heart disease, diabetes, sleep apnea and cancer [4]. In most LMICs, the distribution of childhood nutritional diseases is shifting from a predominance of undernutrition to a dual burden of under-and overnutrition such as obesity [1, 5]. Zanzibar is part of the United Republic of Tanzania, while there is a decline in undernutrition, the prevalence of overnutrition such as overweight/obesity among children is increasing more rapidly. Data from the Tanzania National Nutrition Survey of 2018 shows that around 75% of adolescents in Tanzania are of normal nutritional status. However, above 10% of girls aged 15 to 19 years are underweight, and the prevalence of obesity is becoming a public health concern [6]. The Revolutionary Government of Zanzibar is working toward eradication of all forms of malnutrition, thus reducing overall morbidity and mortality associated with poor nutrition status.

Poor diet quality among school-aged children and adolescents are among the contributing factors to malnutrition in Tanzania and other developing countries [7, 8]. Young children and adolescents gain approximately 20% to 25% of their height and up to 50% of their weight during this age. To support rapid physical growth, there is a need for good nutrition for increased demand of energy, protein, minerals and vitamins [9]. For school children and adolescents aged 5–18 years, Zanzibar Food Based Dietary Guidelines (FBDG) recommends healthy snacks, fruits and vegetables on daily basis [10]. However, school-aged children and adolescents normally rely on the consumption of unhealthy foods such as sweetened beverages and unhealthy snacks due to lack of diet diversity and unhealthy school environment [11, 12]. A recent study conducted in Zanzibar revealed that few adolescents aged 10–17 years consumed iron-rich vegetables, meat, eggs and less than 40% consumed fruits on daily basis [13]. In addition, a cross-sectional study in the Kilimanjaro region found that school children’s diets consisted mainly of cereal-legume meals with low intake of animal-source foods, fruits, and vegetables [14]. Consumption of meals away from home, increased exposure to advertisements that encourage and promote consumption of unhealthy foods, and ease of access to fast foods from school environment, are all likely to be major concerns among school-aged children and adolescents [15].

The associations between diet quality and nutritional status of school-aged children and adolescents have been studied in various countries [16–18]. However, most diet-related studies in Tanzania Mainland and Zanzibar have focused on under-five years children [19–22], leaving a knowledge gap in school-aged children and adolescents, especially in age group of 5 to 19 years. In addition, few small scale studies conducted in Zanzibar have only focused on other aspects of child nutrition such as infant and young child feeding and safety of complementary foods, but not the overall diet quality. Whether the diet quality of school-aged children and adolescents translates into nutrition status is not clear in Zanzibar. Understanding this relationship could be useful to inform food and nutrition policy, and propose interventions that focus on improving the diet quality at school and home. In addition, findings from this research will be important to public health experts and help to work towards ending malnutrition by 2030 [23]. This study therefore presents for the first time evidence of the role of children’s quality diet on the nutritional status in Zanzibar by using school health and nutrition survey which was conducted countrywide.

## Methods

### Study design

The present study is based on secondary data collected from the School Health and Nutrition Survey (SHN) conducted in Zanzibar. This was a large nationally representative cross-sectional survey conducted from October 2021 to February 2022 that aimed to collect nationally representative data on the health and nutritional status of school-age children and adolescents in Zanzibar. This survey was implemented in all 5 regions covering 11 districts (surveys domains) of Zanzibar.

### Sample size and sampling technique

A multi-stage sampling method was used to select a representative sample of school-aged children and adolescents aged 5 to 19 years old in both primary and secondary schools in Zanzibar. A SHN survey was designed to collect information from 93 primary and secondary schools containing school-aged children and adolescents of 5–19 years old in Zanzibar. The recruitment of schools also considered their geographical location to avoid clustering in one area of Zanzibar. A total of 2,604 primary and secondary school-aged children and adolescents were targeted and about 2,556 (98.2%) were successfully interviewed. Consent/assent forms were given to the children to take home for their parents to complete. Only those whose parents consented were recruited for this study, and only after they had also assented.

### Diet quality determination

Diet quality was assessed using Prime Dietary Quality Score (PDQS), an index that assesses the consumption of healthy and unhealthy food groups associated with health and diseases among adults and children [16, 24]. Previously, PDQS has been used to study diet quality among various populations in Tanzania Mainland [25–27]. Foods consumed by children were categorized into 21 food groups, 13 of which are healthy and 8 unhealthy food groups. As used by other researchers, the healthy food groups were dark leafy green vegetables, cruciferous vegetables, carrots, other vegetables, whole citrus fruits, other whole fruits, legumes, nuts, poultry, fish, eggs, whole grains, and liquid vegetable oils [18, 26]. The unhealthy food groups were red meat, cooked potatoes, processed meat, whole milk dairy, refined grains and baked goods, sugar-sweetened beverages, fried foods obtained away from home, and desserts and ice cream [28]. Food groups responses were ranked on a six categories as: 0 = never, 1 = once in a week, 2–3 times a week, 4–6 times a week, once every day and twice per day. Points were assigned for consumption of healthy food groups as follows: 0–1 serving/week, 0 points; 2–3 servings/week, 1 point; and ≥4 servings/week, 2 points. Scoring was revised for unhealthy food groups was as follows: 0–1 serving/week, 2 points; 2–3 servings/week, 1 point; and ≥4 servings/week, 0 points [24]. Points for each food group were then summed to give an overall score. The total PDQS scores ranges from 0 to 42 points. Finally, the total PDQS scores were divided into quintiles, quintile 1 (Q1) indicates the poorest dietary quality and quintile 5 (Q5) indicates the highest [16].

### Anthropometric measurements

Direct measurements of weight (in kilogram) and height (in centimeter) were used to calculate body mass index (BMI) (kg/m^2^). Height was measured with shoes removed, using a Seca 213 portable stadiometer (Seca, Hamburg, Germany). The measurement was repeated, and the average was used for analyses (a third measurement was obtained if the first two measurements were greater than 0.5 cm apart, and the average of the two closest measurements was used in analyses). We used the WHO AnthoPlus package (version 1.0.4) macros file for STATA SE to determine body mass index z-scores (BMIZ) according to age and sex [29]. Children and adolescents were categorized using the WHO cut-off points; thinness <-2 SD, normal between -2SD and +1SD, overweight >+1SD and <+2SD, obese ≥ +2SD from the median value of WHO’s 2006 reference data [29]. We also excluded 154 children who have missing and biologically implausible values.

### Statistical analysis

In this study, categorical variables were presented as proportions and continuous variables as means and standard deviation (Means ± SD). To determine the relationship between diet quality and nutrition status outcomes, i.e thinness, overweight, and obesity, a series of multivariate logistic regression (for dichotomous outcomes) models were built. The PDQS quintiles were used as independent variables of interest. Variables were entered in the multivariate models with outcome variable to identify the adjusted associations by controlling age, gender and residence (rural/urban) as potential confounders. Secondly, body mass index (BMI), the dependent variable of interest, was captured as a continuous variable from components of PDQS. All statistical results were considered significant if *p*<0.05. All analyses were performed using Statistical Package for the Social Science (SPSS) software version 23, Stata software. Also, authors had no access to information that could identify individual participants during or after data collection and analysis.

### Ethical consideration

The study was conducted in line with the Declaration of Helsinki on health research. Ethical approval for the survey was obtained from the Zanzibar Health Research Institute (ZAHRI) under a national research committee that has the legal mandate to provide clearance for health studies in Zanzibar. Permission to start the project was given by the Second Vice Presidents’ Office. Written informed consent was obtained from all children and/or their legal guardian (s).

## Results

### Characteristics of the participants

A total of 2,556 children were analysed in this study. The characteristics of included children are presented in Table 1. There was a slight majority of girls (51.7%) and majority aged between 10 to 14 years (41.4%) with about 53.3% of school-aged children and adolescents living in urban areas. The majority (82.1%) of children had normal weight while 2.6% were classified as being obese. A higher proportion of children (62.6%) are in lower primary level followed by secondary level (30.7%).

**Table 1:** Characteristics of the study participants (N = 2,556).

Characteristics		
	Categories	N (%)
**Age (years)**		
	5 to 9	784(30.6)
	10 to 14	1058(41.4)
	15 to 19	714(27.9)
**Gender**		
	Male	1234(48.3)
	Female	1322(51.7)
**Residence**		
	Rural	1194(46.7)
	Urban	1362(53.3)
**Educational Level**		
	Lower Primary	1601(62.6)
	Upper Primary	167(6.5)
	Secondary	788(30.8)
**PDQS,** *Mean(SD)*		
	Healthy components	*6*.*6(3*.*2)*
	Unhealthy components	*3*.*8(2*.*1)*
**BMI-for-age Z-score** *		
	Thinness (<-2SD)	195(8.1)
	Normal (>+1SD to >-1SD)	1972(82.1)
	Overweight (>+1SD)	173(7.2)
	Obesity (>+2SD)	62(2.6)

*Exclude 154 children whose Height/Weight outside pleusable limit; SD = Standard Deviations

### Diet quality of children

In this study, the mean (SD) score of PDQS was 18.8 (3.2) which ranged from 8 to 33. Overall, school-aged children and adolescents had shown relatively healthy PDQS compared to unhealthy components. The mean (SD) score for the healthy component 6.6(3.2) was higher compared to that of unhealthy component (Table 1). For the healthy food groups of PDQS, consumptions of green leafy vegetables (49.3%), yellow or red fruits (37.8%), legumes (38.3%), fish (36.3%), and vegetable oil (31.5%) were consumed up to three times per week, whereas consumptions of white vegetables (77.3%), cooked vegetables (32.6%), citrus fruits (66.8%), other types of fruits (66.2%), nuts (46.4%), poultry (49.6%), whole grains (61%) and eggs (67.8%) were less than once per week.

Although many healthy foods were reported by school-aged children and adolescents, some of them have reported relying on unhealthy foods. Eating fried foods was a common habit among them and its consumption was reported in 26.3% for up to three times per week, and 21.4% up to four times per week. High consumption of cooked potatoes and roots was reported by the majority (34.7%). More than 20% of children ate refined cereals up to three times per week. In addition, most of the children (31.5%) reported consuming sweets and ice-cream up to three times in the past week, while 16.3% of them consumed sweetened beverages up to three times in the previous week (Table 2).

**Table 2:** Response from components of the Prime Diet Quality Score (PDQS) of school-aged children and adolescents (N = 2,556).

PDQS items	Less than once	Once a week	2–3 times a week	4–6 times a week	Once every day	More than 2 per day
	n(%)	n(%)	n(%)	n(%)	n(%)	n(%)
Green leafy vegetables	484(18.9)	596(23.3)	1259(49.3)	161(6.3)	26(1.0)	30(1.2)
Cabbage leafy vegetables (white or red cabbages)	1977(77.3)	294(11.5)	239(9.4)	26(1.0)	14(0.5)	6(0.2)
Vegetables or fruits that are yellow or red	245(9.6)	349(13.7)	965(37.8)	597(23.4)	178(7.0)	222(8.7)
Any other types of cooked vegetables	834(32.6)	512(20.0)	699(27.3)	382(14.9)	80(3.1)	49(1.9)
Citrus fruits (orange, lemon)	1708(66.8)	402(15.7)	339(13.3)	75(2.9)	25(1.0)	7(0.3)
Any other kind of fruits	1692(66.2)	397(15.5)	367(14.4)	51(2.0)	32(1.3)	17(0.7)
Legumes (beans, peas etc)	524(20.5)	913(35.7)	979(38.3)	96(3.8)	22(0.9)	22(0.9)
Nuts	1186(46.4)	625(24.5)	558(21.8)	130(5.1)	40(1.6)	17(0.7)
White meat (chicken, pigeon etc)	1267(49.6)	778(30.4)	444(17.4)	44(1.7)	15(0.6)	8(0.3)
Fish	124(4.9)	277(10.8)	928(36.3)	842(32.9)	203(7.9)	182(7.1)
Whole grain cereals	1560(61.0)	399(15.6)	347(13.6)	163(6.4)	75(2.9)	12(0.5)
Liquid vegetable oil	793(31.0)	488(19.1)	805(31.5)	384(15.0)	78(3.1)	8(0.3)
Cooked roots and tubers	722(28.2)	720(28.2)	888(34.7)	143(5.6)	26(1.0)	7(0.3)
Red meat	1666(65.2)	538(21.0)	291(11.4)	45(1.8)	13(0.5)	3(0.1)
Canned Meat	2387(93.4)	91(3.6)	60(2.3)	11(0.4)	5(0.2)	2(0.1)
Refined cereals	594(23.2)	351(13.7)	567(22.2)	703(27.5)	148(5.8)	193(7.6)
Sweet beverages	1440(56.3)	567(22.2)	417(16.3)	106(4.1)	22(0.9)	4(0.2)
Fried Foods	749(29.3)	428(16.7)	672(26.3)	546(21.4)	102(4.0)	14(0.5)
Sweets, snacks and Ice-cream	634(24.8)	431(16.9)	806(31.5)	525(20.5)	145(5.7)	15(0.6)
milk or milk products (eg yoghurt)	1626(63.6)	410(16.0)	378(14.8)	97(3.8)	38(1.5)	7(0.3)
Eggs	1732(67.8)	454(17.8)	328(12.8)	25(1.0)	16(0.6)	1(0.0)

### Association between diet quality and nutrition status

In Table 3, a regression analysis was conducted to predict BMI from components of the PDQS and the results indicate that PDQS was statistically associated with BMI (*p*<0.005). For each unit increase in PDQS score, there is a reduction in BMI among school-aged children and adolescents. Similarly, for healthy PDQS component, there was a significant negative association between PDQS and BMI. In terms of food groups, results showed that for each increase in the consumption of green vegetables (*B = -0*.*34*, *p<0*.*001)*, white vegetables(*B = -0*.*52*, *p<0*.*001)*,legumes (*B = -0*.*29*, *p = 0*.*001)*, nuts (*B = -0*.*17*, *p = 0*.*022)*, white meat (*B = -0*.*24*, *p = 0*.*008)*, whole grain cereals (*B = -0*.*14*, *p = 0*.*048)*, cooked roots and tubers (*B = -0*.*26*, *p = 0*.*002)*, citrus fruits (*B = -0*.*61*, *p<0*.*001)*, there was a significant decrease in BMI of school-aged children and adolescents. On the other hand, for each unit increase in consumption of liquid oil (*B = 0*.*14*, *p = 0*.*048)*, cooked vegetables (*B = 0*.*13*, *p = 0*.*038)*, and refined cereals (*B = 0*.*12*, *p = 0*.*023)*, there was a significant increase in BMI.

**Table 3:** Linear regression between PDQS and Body Mass Index (BMI) of school-aged children and Adolescents in Zanzibar.

Variable	*B*	95% Confidence Interval		*P*-value
		Lower	Upper	
Green leafy vegetables	-0.34	-0.50	-0.18	**<0.001**
Cabbage leafy vegetables (white or red cabbages)	-0.52	-0.72	-0.31	**<0.001**
Vegetables or fruits that are yellow or red	0.01	-0.11	0.13	0.857
Any other types of cooked vegetables	0.13	0.01	0.26	**0.038**
Citrus fruits (orange, lemon)	-0.61	-0.78	-0.44	**<0.001**
Any other kind of fruits	-0.13	-0.29	0.03	0.120
Legumes (beans, peas etc)	-0.29	-0.46	-0.12	**0.001**
Nuts	-0.17	-0.32	-0.03	**0.022**
White meat (chicken, pigeon etc)	-0.24	-0.43	-0.06	**0.008**
Fish	-0.01	-0.14	0.13	0.914
Whole grain cereals	-0.14	-0.28	0.00	**0.048**
Liquid vegetable oil	0.14	0.00	0.27	**0.048**
Cooked roots and tubers	-0.26	-0.42	-0.10	**0.002**
Red meat	-0.18	-0.38	0.02	0.072
Canned Meat	-0.02	-0.37	0.32	0.894
Refined cereals	0.12	0.02	0.23	**0.023**
Sweet beverages	0.11	-0.06	0.27	0.214
Fried Foods	-0.16	-0.29	-0.04	**0.011**
Sweets, snacks and Ice-cream	0.06	-0.07	0.18	0.387
Milk or milk products (eg yoghurt)	-0.30	-0.46	-0.14	**<0.001**
Eggs	-0.50	-0.70	-0.30	**<0.001**
**PDQS (Overall)**	-0.070	-0.120	-0.021	**0.005**
PDQS-Healthy component	-0.07	-0.12	-0.02	**0.004**
PDQS-Unhealthy component	0.004	-0.08	0.07	0.895

Furthermore, the logistic regression analysis was conducted to check the risk of thinness, overweight and obesity from diet quality of school-aged children and adolescents. Results show that children with higher PDQS were less likely to have obesity. Children in the third quintile (Q3) had lower odds of obesity (AOR = 0.2, 0.04–0.89 95% CI, *p* = 0.035) compared to the first quintile (Q1). There was no significant association of PDQS with other types of nutrition status such as thinness, normal, and overweight (Table 4).

**Table 4:** Multivariate logistic regression analysis between BMI categories and PDQS quintiles of school-aged children and adolescents in Zanzibar.

Variable	Thinness			Normal			Overweight			Obesity		
	AOR	95% CI	*P*-value	AOR	95% CI	*P*-value	AOR	95% CI	*P*-value	AOR	95% CI	*P*-value
**PDQS quintile**												
QI	Ref	1.0		Ref	1.0		Ref	1.0		Ref	1.0	
Q2	0.94	(0.60–1.47)	0.798	0.96	(0.71–1.29)	0.774	1.02	(0.66–1.56)	0.943	1.33	(0.70–2.51)	0.387
Q3	1.49	(0.93–2.4)	0.097	0.98	(0.68–1.39)	0.888	0.93	(0.54–1.56)	0.775	**0.20**	**(0.04–0.89)** *	**0.035**
Q4	1.21	(0.75–1.93)	0.421	0.86	(0.62–1.19)	0.376	1.15	(0.72–1.83)	0.549	0.92	(0.42–2.00)	0.842
Q5	1.34	(0.86–2.09)	0.194	1.05	(0.76–1.46)	0.747	0.71	(0.42–1.18)	0.188	0.67	(0.29–1.52)	0.339

AOR = Odds ratio; Ref = reference category; Thinness <-2 SD, normal between -2SD and +1SD, overweight >+1SD and <+2SD, obese ≥ +2S of BMI for age-Z score, Model was adjusted for age, gender and residence (rural/urban);

*p<0.05

## Discussion

This study aimed to investigate the associations between diet quality and nutritional status in large sample of school-aged children and adolescents in Zanzibar. The main finding in this study was that diet quality measured by PDQS was associated with obesity. The high PDQS healthy component had the strongest associations with reduction in BMI of children. This may implies the need for implementation of nutritional intervention programs for school-aged children and adolescents for preventing obesity and associated complications. In comparison, diet quality score of unhealthy food groups was found to be associated with obesity among school-aged children in many other countries [16]. In other research, high quality diet was associated with lower BMI and weight status among children [17]. Similarly in Tanzania, diet quality was inversely associated with the risk of overweight and obesity in women of reproductive age [26].

This study found a significant proportion of children consumed unhealthy foods such as sugar-sweetened beverages and fried foods at least three times per week. Several other studies indicated that these foods might be positively associated with an increase in obesity among school-aged children and adolescents, although the results have been inconsistent [30, 31]. This dietary behavior might be attributed to the influence of an unhealthy school food environment as reported in many places in Tanzania and outside [32]. A previous systematic review conducted among 6–18 years school-aged children in African countries highlighted that urban school environment was among the risk factors for overweight and obesity [33]. This might also be the case in this study as majority of included schools were in urban area.

This study highlights that diet quality among school-aged children and adolescents in Zanzibar was largely characterized by low animal-sourced foods and high intake of fried foods and sweetened beverages. Contrary to our initial hypothesis, we observed that the diet quality of the children and adolescents contained some healthy food groups such as vitamin A rich fruits and vegetables. This finding is in line with previous study conducted among school children in Nigeria [34].

Although the relationship between diet quality and childhood obesity is known, it was not entirely clear which specific aspects of the diet should be improved to reduce obesity. Therefore, we investigated the associations between food groups of the PDQS and BMI to identify if any particular foods were driving the nutritional status of children. Some of the foods were negatively associated with BMI as expected. This includes fruits, vegetables, whole cereals and meat [35]. Several mechanisms may explain why these foods have inverse relationship with weight gain. For-example, fruits have high contents of fiber with low energy density, high nutritional value and high satiating power [36].

On the other hand, some foods such as fried foods, cooked vegetables and refined grains were positively associated with an increase in BMI. In comparison, consumption of fried foods and snacks by school-aged children was associated with higher odds of being overweight and obese in other countries [37]. In Zanzibar, cooked vegetables are normally mixed with coconut milk which is known to contain a lot of energy and calories [38]. Fried foods are rich in fats and energy, and therefore may contribute to overweight and obesity [37]. In addition, diets rich in sugar-sweetened beverages are nutritionally unbalanced since they have high content of added sugar and lower content of fiber, protein and various micronutrients [31]. These may suggest that consuming a diet rich in fats, refined grains and sweetened beverages can increase obesity in young children and adolescents.

In this study, there was no association between diet quality measured by PDQS and other categories of weight status such as thinness. This is consistent with a study conducted in Ethiopia among school-aged adolescents, and concluded that the overall diet quality was not significantly associated with stunting and thinness [39].

This study had some limitations, including recall bias as some children may fail to accurately remember foods consumed in the past 7 days. The observed associations between diet quality and risk of obesity in our study should not be interpreted as a cause and effect relationship due to the cross-sectional nature of the SHN survey. Finally, the PDQS has not been validated in Tanzania or other low-income countries. Further research is required to better understand the applicability of the PDQS in Tanzania and Zanzibar.

The strengths of this research are that the PDQS was developed and analyzed on a large representative sample of children. In addition, this is the first study in Zanzibar Island that managed to demonstrate the role of diet quality among school-aged children and adolescents. Moreover, this study has managed to characterize diet quality and weight status in Zanzibar. This may provide actual guidance when designing a potential and high impact intervention package for controlling obesity among children and adolescents.

## Conclusion

It can be conclude that high diet quality was associated with reduction of obesity in a large representative sample of children and adolescents (5–19 years) in Zanzibar. Diet quality measured by PDQS was significantly associated with BMI among school-aged children and adolescents. Furthermore, consumption of healthy foods was negatively associated with BMI, while consumption of unhealthy foods such as fried foods and refined grains was positively associated with BMI. Therefore, improving the dietary quality such as consumption of healthy diet at school and home may help to reduce malnutrition and ultimately reduce health problems among school-aged children and adolescents. It is recommended that future research should consider to use Prime Diet Quality Scores (PQDS) to assess the associations between diet quality with nutritional status of children in Zanzibar.

## Supporting information

S1 Checklist STROBE statement—Checklist of items that should be included in reports of *cross-sectional studies*. (DOCX)

S1 File (XLS)

## Abbreviations

AOR: Adjusted Odd Ratio
BMI: Body Mass Index
PDQS: Prime Diet Quality Score
SD: Standard Deviation
WHO: World Health Organisation
