# Supplementary material for: Influence of diet quality on nutritional status of school-aged children and adolescents in Zanzibar, Tanzania
Source: PLoS One. 2023 Oct 20;18(10):e0293316. doi: 10.1371/journal.pone.0293316 (PMC10588882; doi:10.1371/journal.pone.0293316)
Supplement: S1 Checklist — (DOCX) [file pone.0293316.s001.docx]

STROBE Statement—Checklist of items that should be included in reports of ***cross-sectional studies***

|  | **Item No** | **Recommendation** | **Page No** |
| --- | --- | --- | --- |
| **Title and abstract** | 1 | (*a*) Indicate the study’s design with a commonly used term in the title or the abstract  “***The study design was mentioned in the abstract as a cross-sectional study design based from a nationally representative survey***” | 2 |
|  |  | (*b*) Provide in the abstract an informative and balanced summary of what was done and what was found  “**The abstract highlight the methods and description of the results**” | 2-3 |
| **Introduction** | | | |
| Background/rationale | 2 | Explain the scientific background and rationale for the investigation being reported  “***The background section is present in the manuscript highlight the gap in the literature, the need for this study, problem statement and justification***” | 3-5 |
| Objectives | 3 | State specific objectives, including any pre specified hypotheses  “***This aims to examine the diet quality and its relationship with the nutritional status of school-aged children and adolescents in Zanzibar***” | 2 |
| **Methods** | | | |
| Study design | 4 | Present key elements of study design early in the paper  “***The present study is based on secondary data collected from the School Health and Nutrition Survey (SHN) conducted in Zanzibar. This was a large nationally representative cross-sectional survey conducted from October 2021 to February 2022***” | 5 |
| Setting | 5 | Describe the setting, locations, and relevant dates, including periods of recruitment, exposure, follow-up, and data collection  “***This was a large nationally representative cross-sectional survey conducted from October 2021 to February 2022. This survey was implemented in all 5 regions covering 11 districts (surveys domains) of Zanzibar*** ” | 5 |
| Participants | 6 | Give the eligibility criteria, and the sources and methods of selection of participants  “***All children in the selected school were eligible to be interviewed. A multi-stage sampling method was used to select the participants***” | 5 |
| Variables | 7 | Clearly define all outcomes, exposures, predictors, potential confounders, and effect modifiers. Give diagnostic criteria, if applicable  “***The independent variable(nutrition status) was measured using BMI for age Z-scores using WHO reference data***” | 7 |
| Data sources/ measurement | 8* | For each variable of interest, give sources of data and details of methods of assessment (measurement). Describe comparability of assessment methods if there is more than one group  “***The prevalence and nutritional status was determined using BMIZscores categorised into groups of normal, overweight, unerweight and obesity. Dietary quality was measured using Prime Diet Quality Score (PDQS****)*”. | *6-7* |
| Bias | 9 | Describe any efforts to address potential sources of bias  “***Since this study is a secondary data analysis, during analysis some confounders were controlled to isolate their effects***” | 7 |
| Study size | 10 | Explain how the study size was arrived at  “***Since this was a secondary data analysis, a multi-stage sampling method was used to select a representative sample of school-aged children and adolescents aged 5 to 19 years old in both primary and secondary schools in Zanzibar***”. | 4 |
| Quantitative variables | 11 | Explain how quantitative variables were handled in the analyses. If applicable, describe which groupings were chosen and why  “***Prevalence of Nutrition status and diet quality score were quantitative variables in this study***” | 6-7 |
| Statistical methods | 12 | (*a*) Describe all statistical methods, including those used to control for confounding  “***To determine the relationship between diet quality and nutrition status outcomes, i.e thinness, overweight, and obesity, a series of multivariate logistic regression (for dichotomous outcomes) models were built. The PDQS quintiles were used as independent variables of interest. Variables were entered in the multivariate models with outcome variable to identify the adjusted associations by controlling age, gender and residence (rural/urban) as potential confounders. Secondly, body mass index (BMI), the dependent variable of interest, was captured as a continuous variable from components of PDQS***” | 7 |
|  |  | (*b*) Describe any methods used to examine subgroups and interactions  **NA** |  |
|  |  | (*c*) Explain how missing data were addressed  **NA** |  |
|  |  | (*d*) If applicable, describe analytical methods taking account of sampling strategy  **NA** |  |
|  |  | (*e*) Describe any sensitivity analyses  **NA** |  |
| **Results** | | | |
| Participants | 13* | (a) Report numbers of individuals at each stage of study—eg numbers potentially eligible, examined for eligibility, confirmed eligible, included in the study, completing follow-up, and analysed  **NA** |  |
|  |  | (b) Give reasons for non-participation at each stage  **NA** |  |
|  |  | 1. Consider use of a flow diagram   **NA** |  |
| Descriptive data | 14* | (a) Give characteristics of study participants (eg demographic, clinical, social) and information on exposures and potential confounders  “***The characteristics of school-aged children and adolescents are included in the results-characteristics of the participants (Table 1)”*** | 8 |
|  |  | 1. Indicate number of participants with missing data for each variable of interest   **NA** |  |
| Outcome data | 15* | Report numbers of outcome events or summary measures  NA |  |
| Main results | 16 | 1. Give unadjusted estimates and, if applicable, confounder-adjusted estimates and their precision (eg, 95% confidence interval). Make clear which confounders were adjusted for and why they were included   “***The multivariate logistic regression analysis was adjusted for age, gender and residence (Table 4). They were included as confounders because of their high association with outcome and exposure****”* | 7 |
|  |  | (*b*) Report category boundaries when continuous variables were categorized  “***We used PDQS quintiles, quintile 1 (Q1) indicates the poorest dietary quality and quintile 5 (Q5) indicates the highest***” | 6 |
|  |  | (*c*) If relevant, consider translating estimates of relative risk into absolute risk for a meaningful time period |  |
| Other analyses | 17 | Report other analyses done—eg analyses of subgroups and interactions, and sensitivity analyses  **NA** |  |
| **Discussion** | | | |
| Key results | 18 | Summarise key results with reference to study objectives  “In this study, the mean (SD) score of PDQS was 18.8 (3.2) which ranged from 8 to 33. Overall, school-aged children and adolescents had shown relatively healthy PDQS compared to unhealthy components. The mean (SD) score for the healthy component 6.6 (3.2) was higher compared to that of unhealthy component (Table 1). For the healthy food groups of PDQS, consumptions of green leafy vegetables (49.3%), yellow or red fruits (37.8%), legumes (38.3%), fish (36.3%), and vegetable oil (31.5%) were consumed up to three times per week, whereas consumptions of white vegetables (77.3%), cooked vegetables (32.6%), citrus fruits (66.8%), other types of fruits (66.2%), nuts (46.4%), poultry (49.6%), whole grains (61%) and eggs (67.8%) were less than once per week.  Eating fried foods was a common habit among them and its consumption was reported in 26.3% for up to three times per week, and 21.4% up to four times per week. High consumption of cooked potatoes and roots was reported by the majority (34.7%).  Association between diet quality and nutrition status In Table 3, a regression analysis was run to predict BMI from components of the PDQS and results indicate that consumption of higher diet quality as shown by PDQS was statistically predicted BMI (p<0.005). For each unit increase in PDQS score, there is a reduction in BMI among school-aged children and adolescents. Similarly, for healthy PDQS component, there was a significant negative association between PDQS and BMI. In terms of food groups, results showed that for each increase in the consumption of green vegetables, white vegetables, legumes, nuts, white meat, whole cereals, vegetable oil, cooked roots and tubers, citrus fruits, there is a significant decrease in BMI of school-aged children and adolescents. On the other hand, for each unit increase in consumption of fried foods, cooked vegetables and refined grains there is a significant increase in BMI.  Results show that children with higher PDQS were less likely to have obesity. Children in the third quintile (Q5) had lower odds of obesity (AOR=0.2, 0.04-0.89 95% CI, p=0.035) compared to the first quintile (Q1). There was no significant association of PDQS with other types of nutrition status such as thinness, normal, and overweight. | 8-10 |
| Limitations | 19 | Discuss limitations of the study, taking into account sources of potential bias or imprecision. Discuss both direction and magnitude of any potential bias  “***The limitations are such as recall bias as some children may fail to accurately remember foods consumed in the past 7 days.Also, the PDQS has not been validated in Tanzania or other low-income countries***” | 11 |
| Interpretation | 20 | Give a cautious overall interpretation of results considering objectives, limitations, multiplicity of analyses, results from similar studies, and other relevant evidence  “***The observed associations between diet quality and risk of obesity in our study should not be interpreted as a cause and effect relationship due to the cross-sectional nature of the SHN survey. Further research is required to better understand the applicability of the PDQS in Tanzania and Zanzibar***” | 11 |
| Generalisability | 21 | Discuss the generalisability (external validity) of the study results  **NA** |  |
| **Other information** | | | |
| Funding | 22 Non | “**The author(s) received no specific funding for this work**” | 13 |

*Give information separately for exposed and unexposed groups.

**Note:** An Explanation and Elaboration article discusses each checklist item and gives methodological background and published examples of transparent reporting. The STROBE checklist is best used in conjunction with this article (freely available on the Web sites of PLoS Medicine at http://www.plosmedicine.org/, Annals of Internal Medicine at http://www.annals.org/, and Epidemiology at http://www.epidem.com/). Information on the STROBE Initiative is available at www.strobe-statement.org.
